# Recent developments in the field of radiotherapy for the management of lung cancer

**DOI:** 10.1007/s11604-024-01663-8

**Published:** 2024-09-24

**Authors:** Katsuyuki Shirai, Shuri Aoki, Masashi Endo, Yuta Takahashi, Yukiko Fukuda, Keiko Akahane, Atsushi Musha, Harutoshi Sato, Masaru Wakatsuki, Hitoshi Ishikawa, Ryohei Sasaki

**Affiliations:** 1 Department of Radiology, Jichi Medical University Hospital, 3311-1, Yakushiji, Shimotsuke-shi, Tochigi 329-0498 Japan; 2 Department of Radiology, Jichi Medical University Saitama Medical Center, Saitama, Saitama Japan; 3 QST Hospital, National Institutes for Quantum Science and Technology, Anagawa, Chiba Japan; 4 Gunma University Heavy Ion Medical Center, Maebashi, Gunma Japan; 5 Division of Radiation Oncology, Kobe University Graduate School of Medicine, Kobe, Hyogo Japan

**Keywords:** Lung cancer, Radiotherapy, Particle therapy, SBRT, IMRT

## Abstract

Lung cancer has a poor prognosis, and further improvements in outcomes are needed. Radiotherapy plays an important role in the treatment of unresectable lung cancer, and there have been recent developments in the field of radiotherapy for the management of lung cancer. However, to date, there have been few reviews on the improvement in treatment outcomes associated with high precision radiotherapy for lung cancer. Thus, this review aimed to summarize the recent developments in radiotherapy techniques and indicate the future directions in the use of radiotherapy for lung cancer. Stereotactic body radiotherapy (SBRT) for unresectable stage I lung cancer has been reported to improve local control rates without severe adverse events, such as radiation pneumonitis. For locally advanced lung cancer, a combination of chemoradiotherapy and adjuvant immune checkpoint inhibitors dramatically improves treatment outcomes, and intensity-modulated radiotherapy (IMRT) enables safer radiation therapy with less frequent pneumonitis. Particle beam therapy, such as carbon-ion radiotherapy and proton beam therapy, has been administered as advanced medical care for patients with lung cancer. Since 2024, it has been covered under insurance for early stage lung cancer with tumors ≤ 5 cm in size in Japan. In addition to chemotherapy, local ablative radiotherapy improves treatment outcomes in patients with oligometastatic stage IV lung cancer. A particular problem with radiotherapy for lung cancer is that the target location changes with respiratory motion, and various physical methods have been used to control respiratory motion. Recently, coronavirus disease has had a major impact on lung cancer treatment, and cancer treatment during situations, such as the coronavirus pandemic, must be performed carefully. To improve treatment outcomes for lung cancer, it is necessary to fully utilize evolving radiotherapy modalities, and the role of radiotherapy in lung cancer treatment is expected to increase.

## Introduction

Lung cancer is one of the leading causes of cancer-related death worldwide [1], and improvements in treatment outcomes are warranted. Previously, surgery was considered the mainstay of treatment, and radiation therapy was a treatment option when surgery was not possible. However, there have been remarkable developments in radiotherapy technology in recent years, including the use of stereotactic body radiation therapy (SBRT) for early stage lung cancer, intensity-modulated radiotherapy (IMRT) for advanced-stage lung cancer, and particle beam therapy, leading to improved treatment outcomes. Chemotherapy has been the only treatment for stage IV disease; however, in recent years, aggressive local treatment using radiotherapy has been expected to improve the prognosis of oligometastatic disease. This review aimed to summarize developments in radiotherapy technology and corresponding improvements in treatment outcomes.

## SBRT for early stage lung cancer

Lobectomy is the standard treatment for stage I non-small-cell lung cancer (NSCLC). If the tumor is ≤ 2 cm with a consolidation-to-tumor ratio of ≥ 0.5, segmentectomy becomes the standard treatment from the results of JCOG0802/WJOG4607L [2]. Wedge resection or other sublober resection are considered less-invasive treatment options. However, an increasing number of patients are unable to undergo surgery because of age or comorbidities; therefore, the development of minimally invasive and effective treatment modalities is required [3]. SBRT plays an increasingly important role in improving the local control (LC) of tumors and reducing complications by focusing on high radiation doses with high accuracy.

Prior to the widespread use of SBRT, patients with inoperable stage I NSCLC were treated with the conventional radiotherapy. Two randomized trials, SPACE and TROG 0902 CHISEL, compared the conventional radiotherapy with SBRT in inoperable patients [4, 5]. The TROG 0902 CHISEL trial compared the conventional radiotherapy (66 Gy in 33 fractions or 50 Gy in 20 fractions) with SBRT (54 Gy in 3 fractions or 48 Gy in 4 fractions). The 2-year LC and overall survival (OS) rates were significantly higher in the SBRT group (2-year LC: 86% vs 69%; 2-year OS: 77% vs 59%). Adverse events (AEs) with severity rated ≥ grade 3 occurred in 6% of patients in the conventional radiotherapy group and 12% in the SBRT group [5]. There was no significant difference in respiratory function between the two groups despite the higher biologically effective dose delivered in the SBRT group [6]. Based on the results of these two randomized controlled trials, SBRT is considered the standard treatment for patients with inoperable stage I NSCLC.

Phase II SBRT studies for inoperable stage I NSCLC included RTOG 0236 and JCOG 0403. RTOG 0236 (54 Gy in 3 fractions) revealed that the rates of 3-year LC and 3-year OS were 97.6% and 55.0%, respectively, and that of ≥ grade 3 AEs was 16.3% [7]. In JCOG 0403 (48 Gy in 4 fractions), stage IA patients were classified into the operable and inoperable groups. Among inoperable patients, the 3-year LC and OS rates were 88% and 59.9%, respectively, and that of ≥ grade 3 AEs was 10.6% [8]. No grade 5 AEs were observed in any trial. The results of these trials have led to the recommendation of SBRT as an alternative treatment for patients with inoperable stage I NSCLC.

The favorable outcomes of SBRT in inoperable patients with stage I NSCLC have raised interest in whether SBRT can be indicated for medically operable patients who prefer noninvasive treatment. Randomized control trials comparing surgery and SBRT in patients with operable stage I NSCLC included the ROSEL and STARS trials, which were terminated because of poor patient accruals. A pooled analysis of these two trials revealed that the 3-year OS was significantly better in the SBRT group (79% vs 95%, *P* = 0.037); however, there was no significant difference in the 3-year recurrence-free survival rates between the two groups (80% vs 86%). AEs ≥ grade 3 were observed in 44% patients in the surgery group and 10% of patients in the SBRT group [3]. However, the sample size of this combined analysis was too small to draw definitive conclusions. A propensity score-based analysis of 823 patients ≥ 65 years of age who underwent SBRT, lobectomy, or sublobar resection for stage I NSCLC found that lobectomy was associated with better OS than SBRT (hazard ratio: 0.54, *P* = 0.026), but the difference between SBRT and sublobar resection was not significant (hazard ratio: 0.87, *P* = 0.707) [9]. Prospective phase II trials of SBRT for stage I NSCLC involving operable patients included JCOG 0403 and RTOG 0618. In JCOG 0403, the 3-year OS and LC rates were 76% and 86%, respectively, in operable patients [8]. RTOG 0618 (54 Gy in 3 fractions) showed that the rates of 4-year OS and LC were 56% and 96%, respectively, and that of ≥ grade 3 AEs was 8% [10]. Currently, the STABLE-MATES (NCT02468024) trial comparing SBRT with sublobar resection and the VALOR trial comparing SBRT with anatomical resection (lobectomy or segmentectomy) (NCT02984761) are ongoing, and their results are awaited.

Although there are some differences in the dose fractionation and prescription used in SBRT for stage I NSCLC, higher doses potentially lead to improved LC [11, 12]. Currently, the mainstream method is to match the prescribed dose line to the planning target volume, with higher doses in the center, such that the prescribed dose is 60–90% of the central dose. The JCOG1408 randomized phase III trial comparing 42 Gy in four fractions and 55 Gy in four fractions in patients with stage IA is ongoing [13].

Advances in radiotherapy techniques have established the efficacy and safety of SBRT for stage I NSCLC, making it an effective, noninvasive alternative to surgery, especially for inoperable patients. The use of SBRT for early stage NSCLC has increased and is associated with decreased mortality [14, 15]. In contrast, surgery offers advantages, such as the availability of a pathological diagnosis and ease of determining treatment efficacy. Sharing the advantages and disadvantages of each treatment option and allowing medical professionals to consult patients to determine what is considered optimal will lead to the widespread use of SBRT for stage I NSCLC.

## IMRT for locally advanced lung cancer

**Fig. 1:**
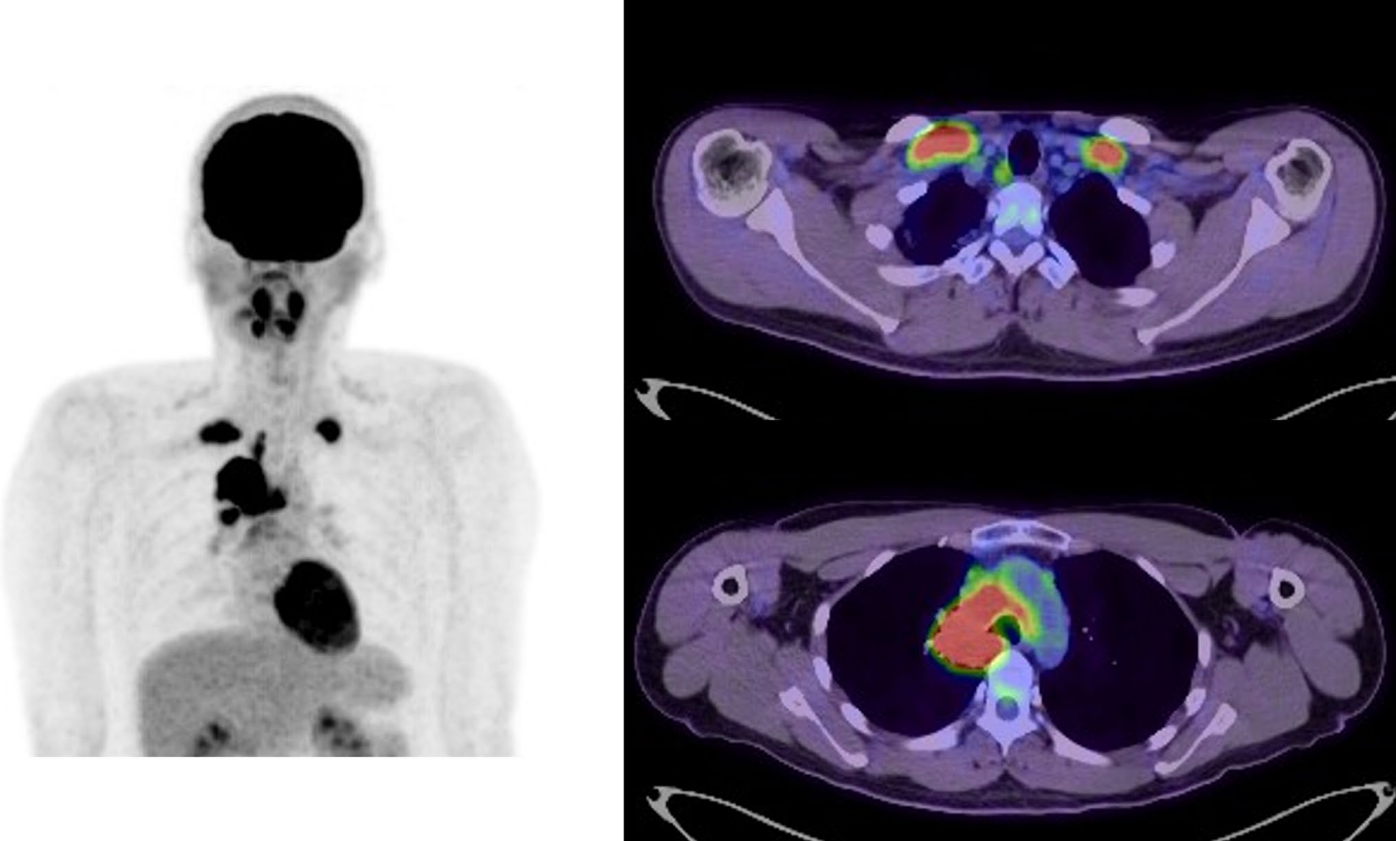
A case of right upper lobe lung cancer (cT3N3M0, stage IIIC). Fluorodeoxyglucose positron emission tomography/computed tomography revealed metastases in the right pulmonary hilum, right mediastinal lymph nodes, and bilateral subclavian lymph nodes

**Fig. 2:**
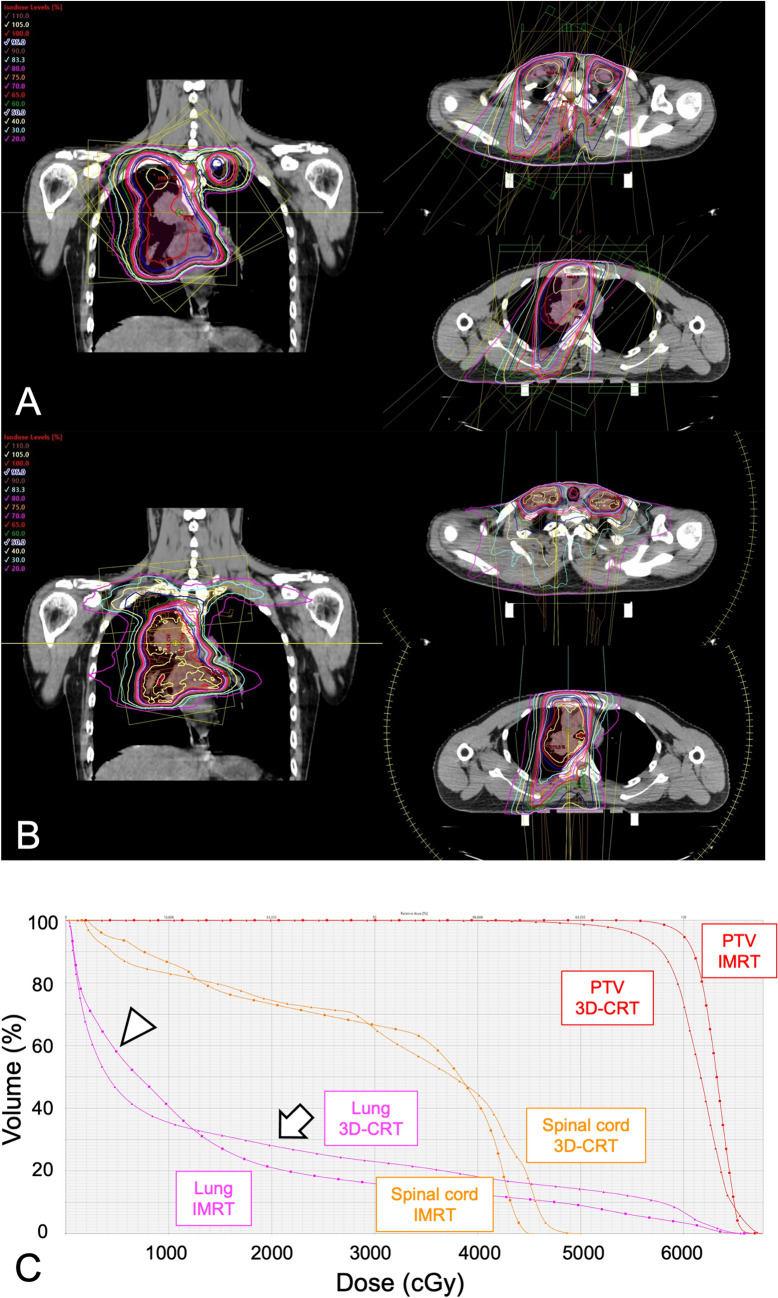
**A** Dose distribution curves of the 3D-CRT plan; 60 Gy is delivered to the isocenter. **B** Dose distribution curves of the IMRT plan; 60 Gy is delivered to 95% of the PTV. **C** Dose-volume histograms of 3D-CRT (▲) and IMRT (■). Doses for the PTV (red), lung (purple), and spinal cord (orange) are shown. The lung V20Gy, i.e., the percentage of lung volume that received ≥ 20 Gy (arrow), is lower with the IMRT plan than with the 3D-CRT plan (28.1% vs 21.0%). The lung V5Gy, i.e., the percentage of lung volume that received ≥ 20 Gy (arrowhead), is higher with IMRT than with 3D-CRT, but the parameter is within acceptable limits (58.9% vs 45.8%). The maximum dose to the spinal cord is 49.9 Gy in the 3D-CRT plan and 45.4 Gy in the IMRT plan. *3D-CRT* three-dimensional conformal radiation therapy; *PTV* planned target volume; *IMRT* intensity-modulated radiotherapy

IMRT has emerged as an effective treatment for locally advanced lung cancer. Previously, the clinical outcomes of unresectable locally advanced lung cancer were poor, and the median survival time of patients treated with radiotherapy alone was only 12 months [16]. Conventional radiotherapy using 60 Gy in 30 fractions combined with chemotherapy has been the standard regimen for unresectable NSCLC. Several studies on radiation dose escalation have been conducted to improve LC. However, RTOG 0617 and RTOG 1106 showed no benefit of high-dose radiotherapy in LC and OS compared with a standard radiotherapy dose of 60 Gy [17, 18]. To improve treatment outcomes, efforts have been made to develop optimal treatment methods that combine radiotherapy and chemotherapy. A randomized phase III trial (PACIFIC study) evaluated the efficacy of durvalumab, an immune checkpoint inhibitor, as consolidation therapy after chemoradiotherapy for unresectable stage III NSCLC, and the median survival time was 47 months [19, 20]. With dramatically improved treatment outcomes, durvalumab consolidation after chemoradiotherapy has become standard treatment. The promising outcomes of durvalumab are considered a possible reason for choosing radiotherapy for locally advanced lung cancer based on real-world data from Japan [21]. Among the AEs associated with chemoradiotherapy reported in the PACIFIC study, radiation pneumonitis was the most frequent AE leading to the discontinuation of durvalumab, and prevention of radiation pneumonitis has become even more important. In a secondary analysis of the RTOG 0617 trial, IMRT was compared with conventional three-dimensional conformal radiation therapy (3D-CRT) [22]. Despite a significantly higher planned target volume in the IMRT group than in the 3D-CRT group, the incidence of ≥ grade 3 radiation pneumonitis in the IMRT group was significantly lower (3.5% vs 7.9%, *P* = 0.039). Based on these results, this study supports the routine use of IMRT for locally advanced NSCLC, which has led to further adoption of IMRT in clinical situations. Various factors are thought to contribute to the reduction in the incidence of severe radiation pneumonitis with IMRT, and the percentage of lung volume that received ≥ 20 Gy (V20Gy) was a particularly significant factor [23]. IMRT can reduce V20Gy compared with 3D-CRT, which is considered a major factor in reducing the risk of radiation pneumonitis with IMRT [24]. In contrast, IMRT tends to have a wider low-dose distribution than 3D-CRT because of its characteristic of delivering irradiation from more diverse angles than 3D-CRT. There are various reports on the potential for low-dose ranges to spread to the lungs and cause severe radiation pneumonitis, and the percentage of lung volume that received ≥ 5 Gy (V5Gy) has been the most studied factor. A multi-center retrospective study in Japan examined risk factors for ≥ grade 2 radiation pneumonitis and found V5Gy to be an important parameter, concluding that V5Gy < 60% is recommended [25]. IMRT planning generally decreases the medium-dose range, such as V20Gy, and increases the low-dose range, such as V5Gy. Planners should be aware of this point and plan IMRT carefully so as not to focus excessively on one parameter at the expense of others. One way to consider this balance is to plan a simulated 3D-CRT and validate that the IMRT plan is superior to the simulated 3D-CRT plan. The treatment planning goals of increasing V5Gy to acceptable levels and decreasing V20Gy compared with that in 3D-CRT are simple and help ensure the quality of IMRT planning. Figure 1 shows a case of lung squamous cell carcinoma primarily in the right lobe with mediastinal and bilateral supraclavicular lymph-node metastases (cT3N3M0, stage IIIC). Figure 2 presents a comparison of the IMRT plan and the simulated 3D-CRT plan in this case.

Elective radiotherapy of the mediastinum has been the standard for stage III lung cancer; however, involved-field radiotherapy, which omits elective radiotherapy, is being considered to reduce adverse events and improve prognosis. A pooled analysis of several studies for stage III NSCLC showed that the involved-field radiotherapy group had a significantly better OS than the elective radiotherapy group [26]. An FDG-PET-based randomized study showed that the risk of locoregional progression in the involved-field radiotherapy group was non-inferior to that in the elective radiotherapy group [27]. These findings suggested that involved-field radiotherapy based with FDG-PET is a promising treatment option for stage III NSCLC.

Another important topic is radiotherapy for limited-stage small-cell lung cancer. The standard treatment has been twice-daily radiotherapy using 45 Gy in 30 fractions combined with chemotherapy; however, the prognosis was poor with MST of 23 months [28]. Although various attempts have been made to improve outcomes of limited-stage small-cell lung cancer, no significant progress has been made in recent decades. A randomized phase III trial compared twice-daily radiotherapy 45 Gy and conventional radiotherapy 66 Gy in 33 fractions and found no significant difference in OS [29]. Recently, the ADRIATIC study, which was a randomized phase III trial, reported that adjuvant immune checkpoint inhibitor after chemoradiotherapy significantly improved OS in 2024 ASCO [30]. It is expected that the treatment regimen for limited-stage small-cell lung cancer will spread in Japan as well.

Recently, cardiac radiation dose has been reported to be associated with mortality risk, and this area has received increasing attention [18, 31]. IMRT can reduce not only the lung dose but also the cardiac dose compared to 3D-CRT; Speirs et al. concluded that IMRT is associated with a significantly lower cardiac dose than 3D-CRT and is associated with cardiotoxic events and prognostic factors [32]. Although no firm conclusions on specific dose-volume parameters or high-risk cardiac exposure sites have been reached, and further data analysis is still needed, it is also important to reduce the cardiac dose as much as possible and avoid radiation-induced cardiotoxicity, which strengthens the rationale for the adoption of IMRT.

In conclusion, recent developments in IMRT for lung cancer have been described, and the adoption and quality improvement of IMRT remain topics of interest. Radiation oncologists should improve their skills in planning IMRT and continue researching the treatment outcomes of IMRT.

## Particle therapies for early and locally advanced lung cancer

Charged-particle therapy involves the use of ionizing radiation, such as protons or carbon ions, and is characterized by an excellent dose concentration, with carbon-ion beam radiotherapy (CIRT) offering the advantage of high biological efficacy [33]. In the treatment of lung cancer, it has advantages over photon radiotherapy in terms of its ability to deliver curative doses while minimizing doses to nearby healthy structures, including the lungs and mediastinal organs, such as the esophagus and heart.

The growing demand for particle therapy for cancer treatment has led to the establishment of institutions for these treatments worldwide. In Japan, proton beam radiotherapy for lung cancer was initiated in 1983, and CIRT was first implemented in 1994 [34]. As of 2024, 26 institutions (19 proton beams, 6 carbon-ion beams, and 1 dual-use beam) are in operation in Japan.

First, a number of reports on early stage lung cancer worldwide, particularly focusing on proton beam radiotherapy, have shown that it reduces toxicity to the lungs and other organs at risk, while maintaining tumor control [35, 36]. In Japan, results of a retrospective analysis of 669 cases of operable/inoperable stage I (UICC 7th edition) NSCLC were reported [37], with a median total irradiation dose of 109.6 BED 10 GyE, 3-year OS of 79.5%, LC of 89.8%, and an incidence of ≥ grade 2 pneumonia of 11.5%, of which grade 3 accounted for 1.7%. Japan has led the clinical development of the CIRT. A domestic multi-center registry analysis of 136 patients with operable stage I NSCLC (UICC 8th edition) reported favorable results, with 5-year OS, cause-specific survival, and LC rates of 81.8%, 91.2%, and 95.8%, respectively, and only 1 case of grade 3 pneumonia (0.7%) [38]. A similar analysis of 95 patients with inoperable stage I NSCLC also showed acceptable 3-year OS, cause-specific survival, and LC rates of 59.3%, 77.1%, and 87.3%, respectively, and a ≥ grade 2 AE rate of 3.2% [39]. The most common schedule used was 60 Gy (relative biological effectiveness [RBE]) in four fractions or 50 Gy (RBE) in one fraction. At present, the superiority of particle therapy in terms of treatment efficacy and survival is not clear; however, it is widely known to be beneficial in reducing normal tissue doses on dose-volume histograms and the risk of AEs, including pneumonia.

**Fig. 3:**
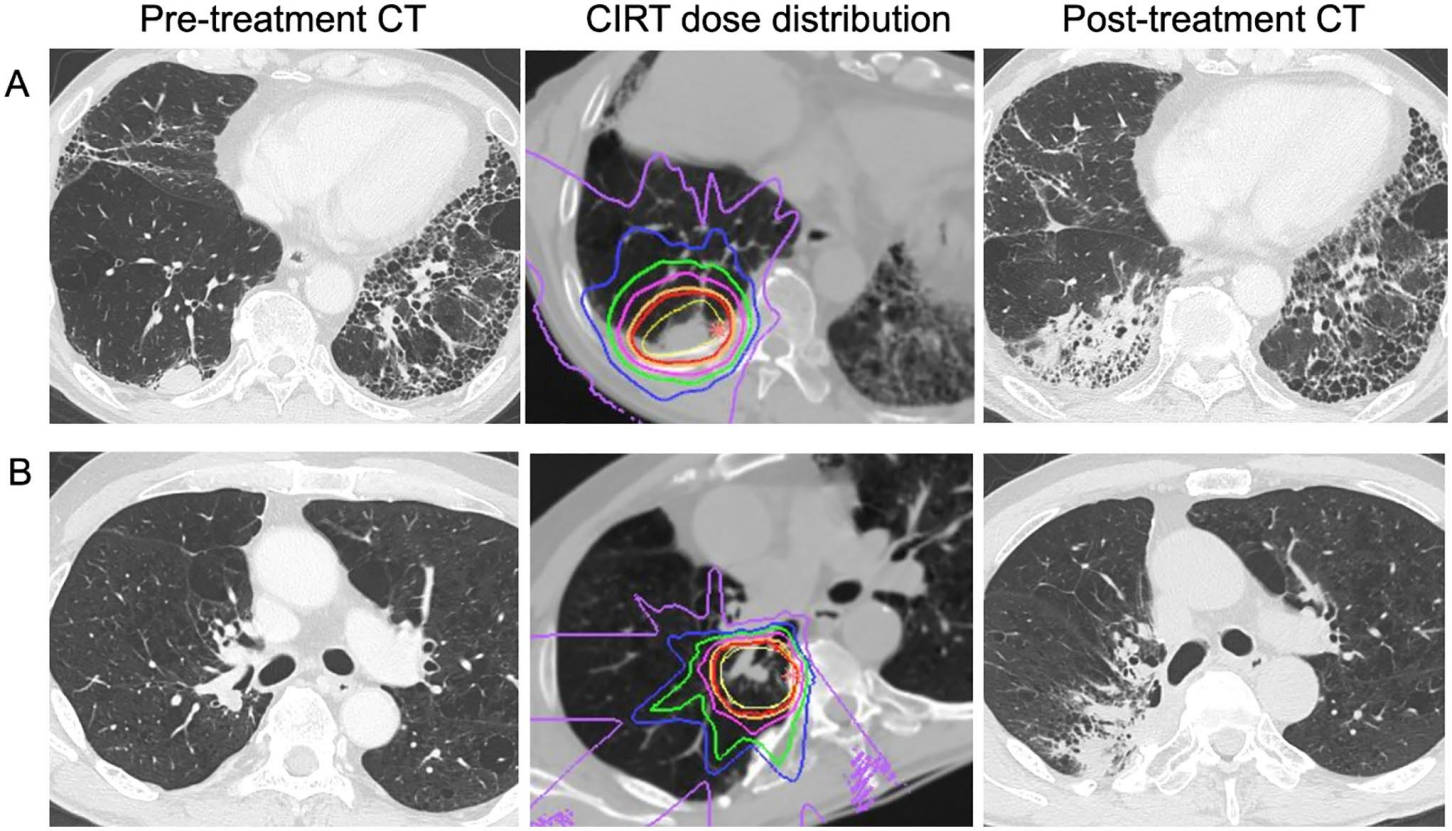
**A** Clinically diagnosed NSCLC (29 mm) with ANCA-associated vasculitis and interstitial pneumonia, receiving with 7.5 mg/day of prednisone (pre-treatment CT, left). The dose fractionation was 50 Gy (RBE) in a single fraction (CIRT dose distribution, middle). The red, yellow, pink, green, blue, and purple lines show 95%, 90%, 70%, 50%, 30%, and 10% doses, respectively; CTV is drawn with the thin yellow line. Radiation pneumonitis was limited to grade 1 (post-treatment CT image, right). **B** Right lower lobe adenocarcinoma (11 mm) with previous right upper-lobe resection (large cell carcinoma, pT2bN0M0) treated with carbon-ion radiotherapy with 68 Gy (RBE) in 12 fractions (protocol for central tumors), requiring treatment for grade 2 pneumonitis. *CT* computed tomography; *CIRT* carbon-ion beam radiotherapy; *NSCLC* non-small-cell lung cancer; *RBE* relative biological effectiveness; CTV ANCA

In addition, its steep and linear dose gradient is expected to provide a safe and curative treatment option, especially for high-risk patient groups receiving photon radiotherapy, such as those with interstitial lung disease-associated lung cancer with a high risk of severe pneumonia and those with central lung cancer with a risk of fatal complications that could affect the mediastinal organs. The typical dose distributions are shown in Fig. 3. In a retrospective analysis of the Japanese National Registry Study on CIRT for early stage NSCLC complicated by interstitial lung disease, only 1 of 30 patients (3%) developed ≥ grade 2 pneumonia [40]. In central lung cancer, dose reductions for mediastinal organs at risk and a reduced risk of fatal AEs have been reported, although only retrospectively, in a small number of 30 cases [41].

In light of these results, Japanese insurance coverage for clinical stage I–IIA (UICC 8th) NSCLC will be available from June 2024, and further data accumulation is expected. At the same time, the benefits of dose distribution are likely to be greater in the case of larger tumors. Therefore, additional data are required to ensure the safety of particle therapy.

Clinical evidence supporting the use of particle radiotherapy in patients with locally advanced NSCLC is also emerging. As a basis for its safety advantage over photon radiotherapy, planning studies have shown dose reductions in organ at risks in locally advanced NSCLC [42], and the possibility of indirectly improving outcomes due to reduced lymphocytopenia has also been suggested [43]. Similar to those for conventional radiotherapy, several trials, including prospective studies, of proton beam radiotherapy have been conducted with concurrent chemotherapy for unresectable stage II–III NSCLC and have shown significant safety benefits [44]. In Japan, a national registry study of 75 patients treated with concurrent chemotherapy reported promising results, with a 2-year OS rate of 73.4% and six cases (8%) of ≥ grade 3 AEs during the follow-up period [45]. Attempts to reduce normal tissue doses further than those of conventional passive irradiation using intensity-modulated proton therapy have also been reported [46]. Current data on CIRT are mainly available for radiotherapy alone in patients who are not candidates for chemoradiotherapy. In Japan, the most common technique is hypofractionated irradiation using dose regimens established in prospective clinical trials, i.e., 64–72 Gy in 16 fractions [47, 48]. A retrospective report showed relatively favorable results, with a 2-year OS rate of 58.7% and ≥ grade 3 AE rate of 4.9% [47]. CIRT offers salvage therapy with potentially acceptable toxicity for patients with locally advanced NSCLC, for whom surgery and chemoradiotherapy are not feasible. However, the future development of concomitant chemotherapy and CIRT is desirable for tumor control.

## Radiotherapy for oligometastatic disease of lung cancer

Oligometastases refer to a condition in which a small number of metastases are present outside the primary tumor. The concept of this disease was proposed by Hellman et al. [49]. Previously, there was no consensus regarding the appropriate definition of oligometastatic status. A consensus report by the European Organisation for Research and Treatment of Cancer (EORTC) was published on oligometastases defining the condition as fewer than five metastases within three organs with local treatment available for all lesions [50]. Reports on oligometastases by the European Society for Radiotherapy and Oncology (ESTRO) and American Society for Radiation Oncology (ASTRO) have also been published, indicating that the number of oligometastases should be limited to those that can be safely treated locally [51]. The growing interest in oligometastases is based on the concept that aggressive local treatments, including surgery or radiotherapy, for distant metastases may achieve long-term disease control in specific subgroups. Oligometastases include synchronous metastases, which are a limited number of metastases observed at the time of initial diagnosis, and metachronous metastases, which are metastases identified after treatment of the primary tumor [52]. Metachronous metastases have been shown to have a better prognosis and to differ in their biological nature [53].

**Fig. 4:**
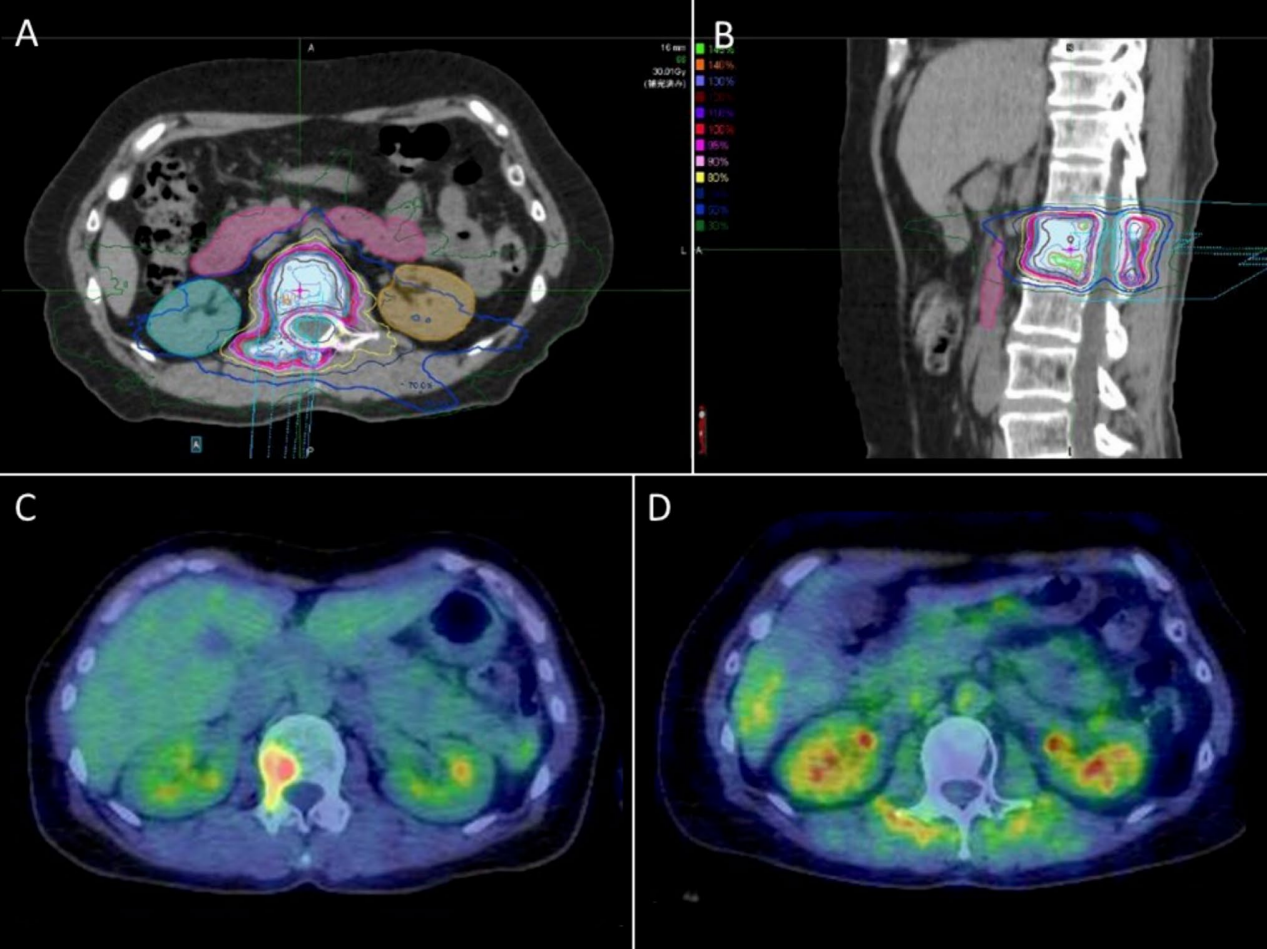
Radiation field and dose distribution of SBRT for oligometastatic bone disease. The dose gradient is made very steep, allowing for a high dose of 24 Gy in 2 fractions while reducing the dose to the spinal cord (**A** and **B**). The accumulation seen on FDG-PET before SBRT (**C**) disappeared at 6 months after SBRT (**D**). *SBRT* stereotactic body radiation therapy; *FDG-PET* fluorodeoxyglucose positron emission tomography

Recently, several randomized phase II trials were conducted for synchronous oligometastases, mainly in lung cancer [54–56]. These studies have shown that local treatment improves overall and disease-free survival without increasing the risk of serious AEs [54–56]. Gomez et al. conducted a multi-center, randomized, phase II trial that enrolled 49 patients with stage IV NSCLC who had up to three oligometastatic lesions and no progression for at least 3 months after initial systemic treatment. One group received maintenance therapy or observation (MT/O) (*n* = 25), and the other received local consolidative therapy (LCT), including surgery, SBRT, and conventional fractionation, in addition to maintenance therapy (*n* = 24). They found a significant difference in median progression-free survival (PFS) (11.9 months with LCT vs 3.9 months with MT/O; *P* = 0.005) [54]. Additionally, subsequent follow-up showed an extension of median survival time in the LCT group compared to in the MT/O group (41.2 months with LCT vs 17.0 months with MT/O; *P* = 0.017) [55]. Iyengar et al. compared a maintenance chemotherapy alone group (*n* = 15) with an LCT group (*n* = 14); SBRT was added as a local treatment to the maintenance therapy. They found a significant difference in median PFS (9.7 months with LCT vs 3.5 months with maintenance; *P* = 0.01) [56]. With advancements in radiation therapy techniques, particularly SBRT, there has been increasing interest to improve outcomes in this condition. In the SABR-COMET trial, which involved patients with up to five oligometastases in three organs and no progression after initial systemic treatment, including 18% of patients with lung cancer (*n* = 99), the group receiving SBRT for all lesions in addition to the standard of care (*n* = 66) was compared with the group treated with the standard of care approach alone (*n* = 33) [57]. A significant difference was observed in the median survival time (41 vs 28 months in the SBRT and control groups, respectively; *P* = 0.09). In this trial, treatment-related deaths occurred in 4.5% of the patients in the SBRT group, indicating the importance of careful patient selection. Furthermore, reports on the efficacy of SBRT for oligometastases in various organs, including the bones [58], lungs [59], adrenal glands [60], and lymph nodes [61], have been published. Figure 4 shows an example of the findings in a patient received SBRT for oligometastatic bone disease. SBRT enables high-dose administration to spinal metastases, while reducing the dose to the spinal cord under tight fixation and image guidance techniques. In the field of lung cancer, Flannery et al. reported a 21% 5-year OS rate after performing stereotactic radiosurgery in patients (*n* = 42) with synchronous solitary brain metastasis within 6 months of initial treatment, including surgery, radiotherapy, and chemoradiotherapy [62]. Additionally, Lin et al. reported a 3-year local PFS of 80.2%, 3-year PFS of 21.9%, and 3-year OS of 45.3% after SBRT in patients with three or fewer oligorecurrent lung lesions without active distant metastases after the initial treatment [63].

The prognosis of advanced NSCLC has significantly improved with the emergence of molecular targeted therapies, such as tyrosine kinase inhibitors, and the introduction of immune checkpoint inhibitors, leading to increased interest in combined therapy with SBRT for oligometastases and these drugs as systemic therapy. For instance, the SINDAS trial showed that the addition of local treatment with radiotherapy post-treatment with a tyrosine kinase inhibitor significantly improved PFS and OS in patients with EGFR mutation-positive NSCLC with synchronous oligometastases [64]. NRG-LU002, a randomized phase II/III trial, evaluated immune checkpoint inhibitors based systemic therapy with or without LCT for oligometastatic NSCLC [65]. In this study, immune checkpoint inhibitors and LCT had a PFS HR of 0.90 with no advantage of OS, indicating that patient selection may optimize this therapeutic ratio. The use of radiotherapy in oligometastatic lung cancer treatment will continue to expand, and further data and high-quality clinical trials are required to elucidate the role of SBRT.

## Emerging radiotherapeutic techniques for a respiratory movement in lung cancer

Respiratory motion hinders the use of radiotherapy as a localized treatment. In recent years, the importance of respiratory motion management has increased with the widespread use of image-guided radiotherapy for the precise alignment of patients. Respiratory motion causes motion artifacts in image acquisition for treatment planning, potentially hindering accurate delineation of targets [66–68]. Additionally, this may lead to an increase in the margin size in treatment planning and blurring of the dose distribution during treatment delivery [66, 69, 70]. Appropriately implementing respiratory motion management can shrink the treatment volume and reduce the radiation dose to the surrounding normal tissues, thereby decreasing AE incidence [71]. The methods used in respiratory motion management in radiation oncology include motion-encompassing, respiratory-gated, breath-holding, forced shallow breathing, and respiration-synchronized techniques [66].

**Fig. 5:**
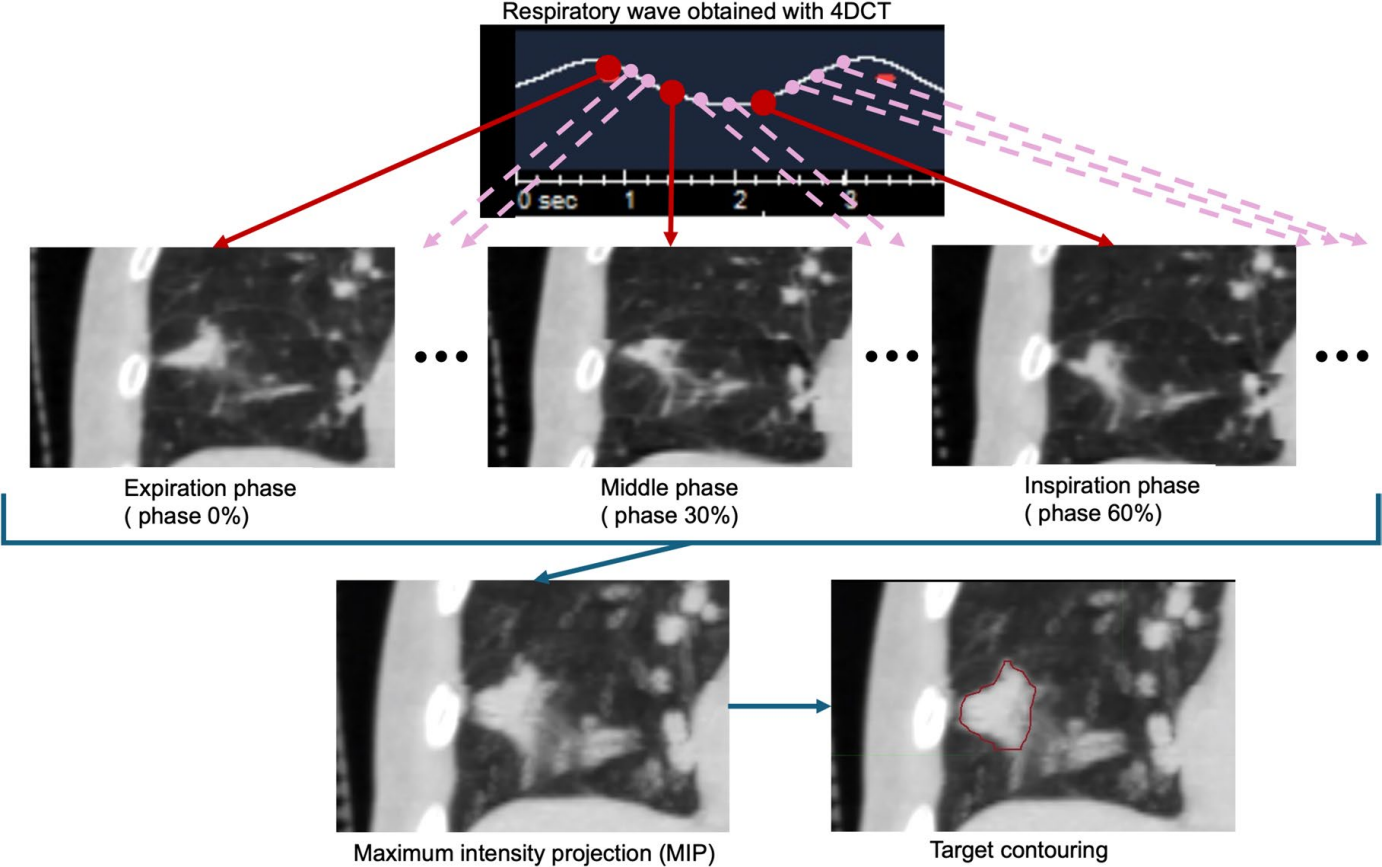
Schema of 4DCT reconstruction, and contouring using 4DCT image dataset. Images at each respiratory phase is reconstructed according to the respiratory wave obtained with CT scanning. *4DCT* Four-dimensional CT

Motion-encompassing methods estimate the average position and range of target motion. These include slow CT scanning, inhaled and exhaled breath-hold CT, and four-dimensional CT (4DCT). In particular, 4DCT is most frequently used in the simulations of thoracic cancer [72]. Typical 4DCT images are shown in Fig. 5. In addition, Korreman et al. reported that the use of 4D image guidance, including 4DCT, for all motion magnitudes in the case of fractionated lung tumor irradiation is highly beneficial, and a 4DCT scan for treatment planning should be used for motion magnitudes of > 8 mm [73]. These acquired image datasets are used to estimate respiratory motion, which is accounted for as part of the internal margin defined in the ICRU report [69], and is used to create an internal target volume (ITV). The limitation is that motion artifacts caused by unstable patient respiration can affect the estimation of the range of the target movement.

Respiratory-gated and breath-hold techniques are used for imaging and treatment delivery. In the former method, a range called “gate” is set in the respiratory cycle, and irradiation is performed only within the gate of the respiratory cycle. In the latter method, irradiation is delivered when respiration stops within the gate. Respiration-synchronized techniques are irradiation methods that track the target in real time by predicting a patient's respiratory cycle, which correlates with the target location. This enables irradiation while maintaining a 100% duty cycle [66]. Because there is a delay between the acquisition of the respiratory signal and the beam on–off, the accuracy of the prediction of the respiratory wave is important in this method.

**Fig. 6:**
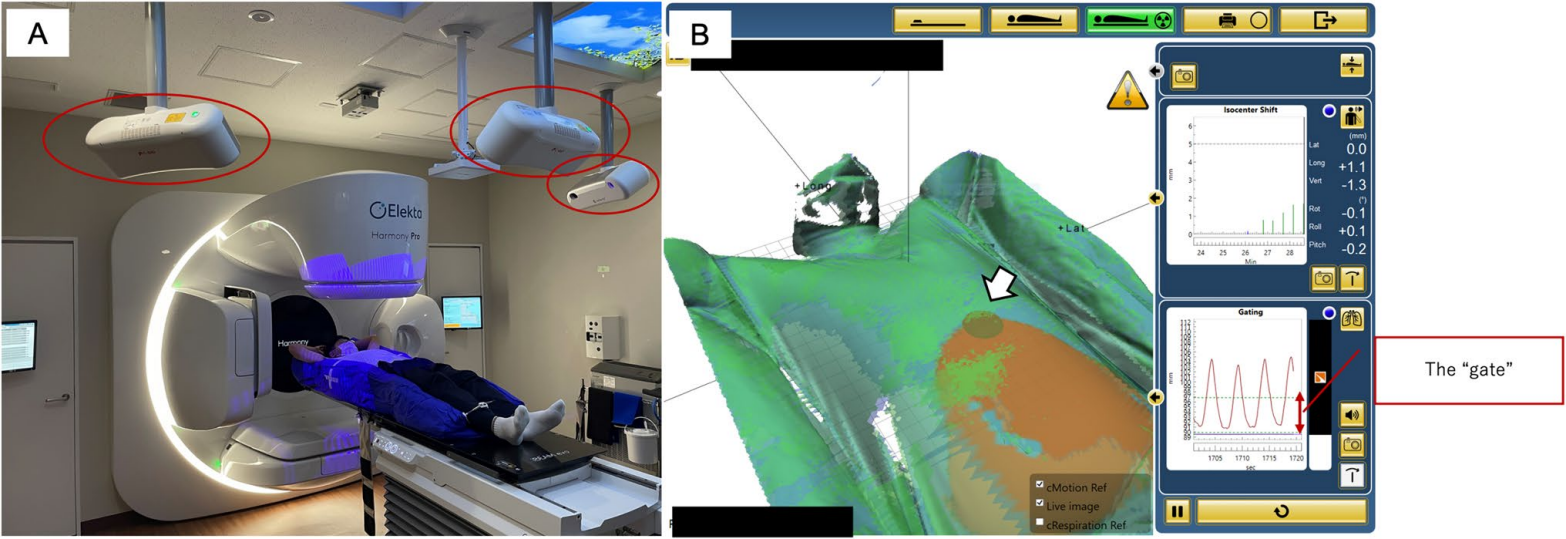
**A** Example of a surface imaging system (C-RAD Catalyst + TM). **B** Surface image during respiratory motion monitoring with the “gate”. Red region spreading on abdomen indicates that the region exceeding the tolerance for patient’s movement. A circle region indicated by white arrow is a region of interest for acquiring the respiratory wave, and the respiratory wave is illustrated in the right-lower section of this figure

To manage the aforementioned respiratory motions, a surrogate marker for the target is often applied. One is an implanted marker, such as a gold marker, near a target in the body, and the other is the patient’s body surface. In the latter method, respiratory movements of the chest and abdomen are conventionally monitored using pressure sensors and infrared light, based on the assumption that there is a correlation with the movements of the target. In recent years, with the availability of body-surface imaging systems that use optical systems to visualize the body surface (Fig. 6), the use of such systems to monitor patient movement and respiratory cycles has become common [72]. Utilizing information from a patient’s body surface is beneficial because the imaging techniques are noninvasive. However, it is necessary to know in advance how the respiratory waves obtained from the body surface correlate with the target location, and whether they are reproducible. Furthermore, when performing respiratory-gated techniques, there are two modes of respiratory gating: phase and amplitude gating. In the case of phase gating, baseline shifts in the respiratory cycleled to target position errors of up to 2.1 mm [74].

Forced shallow-breathing methods are used to restrict diaphragmatic motion by compressing the abdomen. Compression is performed using an adjustable compression plate or a dedicated airbag with a pressure gauge [75], which is a simpler respiratory motion management technique than the others and is often used in conjunction with the ITV technique. Although it has been reported to suppress target movement in many patients, there are cases in which the target movement is unchanged or enhanced [76, 77]. Patient-specific confirmation is important.

In the U.S., the most widely employed techniques in the order of frequency are the breath-hold, forced shallow-breathing, respiratory gated, and respiration-synchronized techniques [72]. When applying these techniques at a particular facility, it is important to know their advantages and disadvantages in advance, because of the characteristics of each method.

## Radiotherapy during the coronavirus disease (COVID-19) pandemic

Coronavirus disease (COVID-19) has resulted in a global pandemic. There are three main routes of transmission: droplets, contact, and aerosols [78, 79]. Aerosol generation is also triggered by physiological events, such as the cough reflex. Owing to the specificity of the disease, patients with lung cancer can generate aerosols through their cough reflex during radiotherapy. These aerosols may contain many salivary components that may contaminate the air and the linear accelerator tabletops around the patient, thereby increasing the risk of infection. Patients can easily be exposed to staff preparing for treatment administration around the radiotherapy equipment, especially if they are not wearing a surgical mask during radiotherapy. Radiotherapy rooms are generally small enclosed spaces. Previous research has confirmed that even normal conversations have a considerably higher potential to cause airborne viral infections in closed environments [80]. Furthermore, aerosolized viral particles can travel distances of up to 6 m [81], which is sufficient to spread throughout a radiation therapy room and cause secondary infections. The survival time of severe acute respiratory syndrome coronavirus-2 (SARS-CoV-2) is also problematic, reported to be 72 h after surface deposition on plastics and stainless steel and 3 h in suspended aerosols [82]. A simulation study provided evidence for areas prone to contamination by patients not wearing a surgical mask in a supine position on a linear accelerator tabletop and by the staff in a real radiotherapy room [83]. Notably, the area surrounding the patients at a 0.7-m radius from their mouth tended to be highly contaminated, and the smaller the contamination particle size, the farther it reached. Staff moving around within this range to position patients are easily exposed to such contaminants. Moreover, the radiotherapy staff working around a patient sometimes have a considerable amount of contaminated particles adhered to the facial area. Since the COVID-19 pandemic, surgical masks have been generally worn by medical staff and patients in radiotherapy facilities [84]. However, it is important to wear personal infection prevention devices, such as surgical masks, face shields, and gowns, when approaching a 0.7-m radius around the patient’s mouth. Decontamination is also necessary, because it is difficult to completely prevent infection through contact with patients. Hand hygiene is a basic infection control measure; since SARS-CoV-2 has an envelope, hand disinfection with alcohol and washing hands with soap and running water are both effective [79]. Alcohol wiping of areas on which viruses have dispersed or adhered, such as radiotherapy tabletops around patients, is also recommended. In recent years, a few studies have reported that mouthwashes containing cetylpyridinium chloride or povidone-iodine can reduce the oral viral load of SARS-CoV-2 [85, 86]. Because it is difficult to completely block aerosol outbreaks, entering the radiotherapy room after using mouthwash for each patient may be a relatively simple method of infection control. During an infectious outbreak, such as the COVID-19 outbreak, it is important to continue cancer treatment while attempting standard precautions to prevent infection among medical staff and patients.

## Conclusion

Advances in radiotherapy techniques, irradiation methods, and appropriate chemotherapy combinations have significantly improved treatment outcomes for lung cancer. Continued efforts are required for further progress, and radiation oncologists will need to make efforts to conduct prospective clinical trials and utilize large-scale real-world data. Multidisciplinary treatments require a team of radiation oncologists, pulmonologists, surgeons, technicians, medical physicists, and nurses.
